# Quercetin improves macrophage reverse cholesterol transport in apolipoprotein E-deficient mice fed a high-fat diet

**DOI:** 10.1186/s12944-016-0393-2

**Published:** 2017-01-14

**Authors:** Yingjie Cui, Pengbo Hou, Fahui Li, Qinghua Liu, Shucun Qin, Guanghai Zhou, Xuelian Xu, Yanhong Si, Shoudong Guo

**Affiliations:** 1 Key Laboratory of Atherosclerosis in Universities of Shandong Province, Institute of Atherosclerosis, Taishan Medical University, 2# Yingsheng East Road, Taian, Shandong Province 271000 China; 2 Research Center on Life Sciences and Environmental Sciences, Harbin University of Commerce, Harbin, 150076 China; 3 Department of Chemistry and Chemical Engineering, Weifang University, Weifang, 261061 China; 4 Affiliated Hospital of Taishan Medical University, Taian, 271000 China; 5 School of Medicine and Pharmacy, Ocean University of China, Qingdao, 266003 China

**Keywords:** Cholesterol, Animal, Diet, Medicine, Atherosclerosis, LC-MS/MS

## Abstract

**Background:**

Quercetin, one of the most widely distributed flavonoids in plants, has been demonstrated to reduce hyperlipidaemia and atherosclerotic lesion formation. Reverse cholesterol transport (RCT) plays a crucial role in exporting cholesterol from peripheral cells, which is one mechanism utilized in the prevention and treatment of atherosclerosis. The aim of this study is to investigate whether quercetin reduces lipid accumulation by improving RCT in vivo.

**Methods:**

Apolipoprotein E-deficient mice fed a high-fat diet were used to investigate the effect of quercetin on RCT by an isotope tracing method, and the underlying mechanisms were clarified by molecular techniques.

**Results:**

These novel results demonstrated that quercetin significantly improved [^3^H]-cholesterol transfer from [^3^H]-cholesterol-loaded macrophages to the plasma (approximately 34% increase), liver (30% increase), and bile (50% increase) and finally to the feces (approximately 40% increase) for excretion in apolipoprotein E-deficient mice fed a high-fat diet. Furthermore, quercetin markedly increased the cholesterol accepting ability of plasma and high-density lipoprotein (HDL) and dramatically decreased the content of malondialdehyde in plasma and oxidized phosphocholine carried by HDL. Therefore, the underlying mechanisms of quercetin in improving RCT may be partially due to the elevated cholesterol accepting ability of HDL, the increased expression levels of proteins related to RCT, such as ATP-binding cassettes (ABC) A1 and G1, and the improved antioxidant activity ﻿of HDL﻿.

**Conclusion:**

Quercetin accelerates RCT in an atherosclerosis model, which is helpful in clarifying the lipid-lowering effect of quercetin.

## Background

The cardiovascular protective effects of the natural product quercetin may be partially attributed to its antioxidant [1] and anti-inflammatory [2] properties. It is noteworthy that recent studies indicated that quercetin decreases hyperlipidaemia in different high-fat diet fed animals [3–6].

Hyperlipidaemia is one of the major risk factors for the onset and progression of atherosclerosis, and reverse cholesterol transport (RCT) is believed to facilitate it by transporting excess cholesterol from peripheral tissues to the liver and small intestine for excretion [7]. Considering its lipid-lowering effects, quercetin may play an important role in the development of atherosclerosis by regulating RCT. An in vitro study has indicated that quercetin may activate ATP-binding cassette (ABC) A1 by up-regulating peroxisome proliferator-activated receptor γ (PPAR-γ) and liver X receptor α (LXR-α) [8], while other research showed that quercetin enhances ABCA1 expression through a p38-dependent pathway in macrophages [9]. Our previous data indicated that quercetin improves the expression of RCT-related proteins in apolipoprotein E-deficient (*apoE* ^*−/−*^) mice [3]. However, there is little known about the in vivo biological effects and mechanisms of quercetin on RCT. Based on the previous reports, we suppose that the lipid-lowering effect of quercetin may be partially attributed to its stimulation of RCT.

The aim of this study is to investigate whether quercetin improves RCT in an atherosclerosis model of “*apoE* ^*−/−*^ mice” fed a high-fat diet and to investigate the underlying mechanisms.

## Methods

### Materials

Raw 264.7 macrophages were purchased from Shanghai BoYao Biological Technology Co., Ltd. (Shanghai, China). Quercetin was a Sigma-Aldrich product. Dulbecco’s modified Eagle’s medium (DMEM) and feotal bovine serum (FBS) were from Gibco (BRL, Gaithersburg, MD, USA). Complete protease inhibitor cocktail tablets were purchased from Roche (Schweiz, Germany). RIPA lysis buffer was a product of Solarbio (Beijing, China). Rabbit polyclonal antibody against ABCG1, mouse monoclonal antibody against ABCA1 and rabbit monoclonal antibody against scavenger receptor B type 1 (SR-B1) were from Abcam (Cambridge, MA, USA). Enhanced chemiluminescence (ECL) kits were purchased from Thermo Scientific Pierce (Rockford, IL, USA). 1-palmitoyl-2-(5′-oxo-valeroyl)-*sn*-glycero-3-phosphocholine (POVPC), 1-palmitoyl-2-(9′-oxo-nonanoyl)-*sn*-glycero-3-phosphocholine [(ALDO)PC], 1-palmitoyl-2-azelaoyl-*sn*-glycero-3-phosphocholine (PAzPC), 1-palmitoyl-2-glutaryl-sn-glycero-3-phosphocholine (PGPC) and 1-hexadecyl-2-azelaoyl-*sn*-glycero-3-phosphocholine [(COOH)PC] were purchased from Avanti Polar Lipids Inc. (Alabaster, AL, USA). An assay kit for malondialdehyde (MDA) was the product of Nanjing Jiancheng Bioengineering Institute (Nanjing, Jiangsu, China). All reagents used in this study were of analytical grade.

### Animals and grouping

Twenty-four *apoE* ^*−/−*^ mice with a C57BL/6 genetic background (male, 20 ± 2 g) were purchased from Beijing HFK Bioscience Co., Ltd. (license number: SCXK2009-0004). All experiments were approved by the Laboratory Animal Ethical Committee of Taishan Medical University and followed the NIH guidelines for the care and use of animals. Mice were fed a high-fat diet (15% fat and 1.25% cholesterol). After a one-week adaptive phase, the mice were randomly divided into 2 groups, namely, the CMCNa group (*n* = 12, 0.5% carboxymethyl cellulose sodium by gavage) and the quercetin group (*n* = 12, 12.5 mg/kg/d quercetin in 0.5% CMCNa by gavage). The dosage of quercetin was determined in accordance with a previous study [3].

### Preparation of high density lipoprotein (HDL)

After 8 weeks of treatment, 6 animals from each group were randomly chosen and blood was sampled from the retro-orbital sinus after 6 h fasting. Plasma was prepared by the centrifugation of the fresh blood at 1100 × g for 15 min at 4 °C. Lipoproteins were prepared using sequential ultracentrifugation according to the method described in a previous publication [10]. Briefly, the plasma density was adjusted to 1.063 g/mL for ultracentrifugation at 10 °C (70,000 rpm for 24 h). The upper layer of non-HDL was removed, and the rest was adjusted to 1.21 g/mL density for ultracentrifugation at 70,000 rpm for another 48 h to obtain HDL in the upper layer. The protein content was determined by the Bradford method. Isolated plasma and HDL were kept under nitrogen gas at 4 °C for in vitro cholesterol efflux assays and liquid chromatography-tandem mass spectrometry (LC-MS/MS) analysis.

### Preparation of [^3^H]-cholesterol-loaded macrophages

[^3^H]-cholesterol (5 μCi/mL) and oxidized low-density lipoprotein (LDL, 100 μg/mL) were mixed and pre-incubated at 37 °C for 30 min. Raw 264.7 macrophages were cultured in DMEM medium (10% FBS) supplemented with the above mixture. The cells were harvested after 48 h incubation and intraperitoneally injected into mice (6.0 × 10^5^ cells containing 4.0 × 10^5^ counts/min in 0.5 mL of DMEM per mouse) [11].

### RCT assay in vivo

After 8 weeks of treatment, the other 6 mice in each group were used for isotope tracing study. After the injection of [^3^H]-cholesterol-loaded macrophages, blood was sampled from the retro-orbital sinus at the time points of 0, 6, 12 and 24 h, 100 μL per mouse each time. Faeces were collected every 24 h. Blood was sampled at 48 h after the injection, as described above. Animals were killed, and the liver and bile were collected.

Liver (0.3 g) and faeces were homogenized in a hexane/isopropanol (3:2, v/v) solution, and the mixtures were placed on a shaker for 12 h at room temperature. Then, the mixture was centrifuged at 5000 × g for 15 min, and the supernatant was kept for measurement. Prepared samples were transferred into 5.0 mL scintillation vials, and 4.0 mL of HIONIC FLUOR™ complete LSC-cocktail (PerKinElmer, Inc., Waltham, MA, USA) was then added and mixed well. The scintillation vials were counted on a SN-6930B type liquid scintillation counter (BECKMAN, USA). The results were expressed as a percentage of injected [^3^H]-cholesterol or directly by counts per minute (CPM).

### The cholesterol efflux assay in vitro

This experiment was performed as suggested by Low et al. with some modifications [12]. Briefly, Raw 264.7 macrophages were plated into 24-well plates at a final density of 1 × 10^5^ cells per well. Six hours later, the culture medium was discarded and cells were gently washed with PBS 3 times. Then, 600 μL of DMEM containing 1% FBS and 1 μCi/mL [^3^H]-cholesterol was added to each well and incubated for another 24 h. After the incubation, the medium containing [^3^H]-cholesterol was removed, and the cells were gently washed with PBS 3 times. Cells were treated with 500 μL of DMEM containing 1% FBS in the presence or absence of quercetin for 4 h, and 100 μL of HDL (final concentration of 20 μg/mL) or 10 μL of plasma was then added and incubated for another 6 h. The culture medium was collected in 1.5-mL microfuge tubes and centrifuged at 14,000 rpm for 10 min at room temperature to remove cellular debris. Cells in the well were treated with 1 mL of hexane/isopropanol (3:2, v/v) for 30 min at room temperature. The extract was transferred into 1.5-mL microfuge tubes and centrifuged as described above. Next, 200 μL of medium or cell extract was used for measuring [^3^H]-cholesterol, as described above.

### Protein isolation, electrophoresis, and western blotting

Total proteins from the cells were extracted and prepared for western blotting, as explained in detail in a previous publication [13].

### Lipid extraction

Lipids from HDL were extracted according to the method of Bligh and Dyer with some modifications [14]. (1) For 100.0 μg of HDL, 1.0 mL of water and 3.75 mL of 1:2 (v/v) CHCl_2_: MeOH were added and vortexed for at least 5 min; (2) 1.25 mL of CHCl_2_ was added and vortexed well; (3) 1.25 mL of water was added and vortexed well. (4) The mixture was centrifuged at 1500 rpm for 10 min, and the bottom organic phase was recovered and dried under nitrogen gas. (5) The dryness was dissolved in 0.5 mL of mobile phase and centrifuged at 20,000 rpm for 20 min, and the upper phase was used directly for LC-MS/MS analysis.

### Measurement of oxidized lipids by LC-MS/MS

The composition of LC-MS/MS and the column used were the same as a previous publication [13]. The mobile phase A was composed of 2.0 mM ammonium acetate and 0.1% acetic acid in methanol, and mobile phase B was composed of 2.0 mM ammonium acetate and 0.1% acetic acid in water. The flow rate was 0.4 mL/min with the following elution gradient: 0.01-25 min: 5% B-1% B; 25–26 min: 1% B-5% B; 26–30 min: 5% B. Quantification was performed with Analyst Software 1.6 (AB SCIEX, USA).

### Data analysis

All of the bioassay results were expressed as the mean ± standard deviation (*SD*) for at least three independent experiments. Statistical analysis was performed using one-way analysis of variance (ANOVA) followed by Tukey’s test. Differences were considered to be significant at a *P* < 0.05.

## Results

### Quercetin improves RCT in *apoE*^*−/−*^ mice fed a high-fat diet

**Fig. 1:**
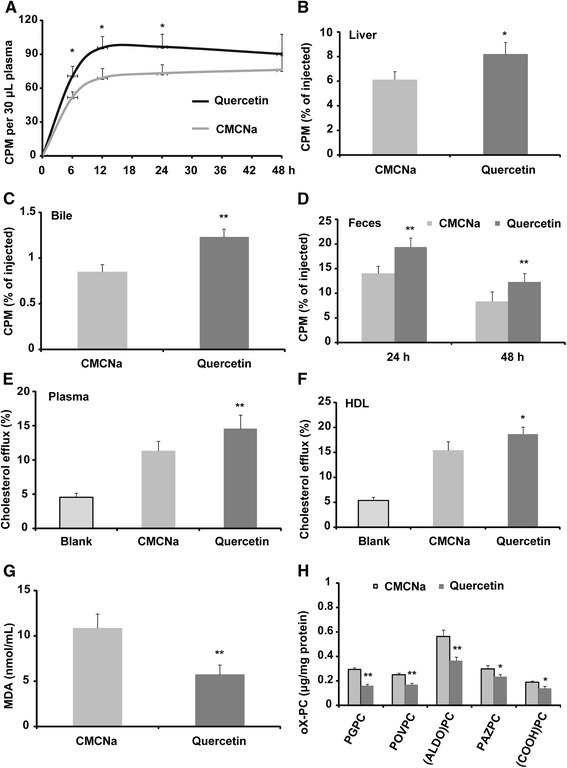
Quercetin improves reverse cholesterol transport in *apoE* ^*−/−*^ mice (*n* = 6). **a** Quercetin increases the transfer rate of [^3^H]-cholesterol from injected foam macrophages to the plasma of *apoE* ^*−/−*^ mice; (**b**) [^3^H]-cholesterol in the liver of the *apoE* ^*−/−*^ mice after 48 h injection; (**c**) [^3^H]-cholesterol in the bile of the *apoE* ^*−/−*^ mice after 48 h injection; (**d**) [^3^H]-cholesterol in the faeces of the *apoE* ^*−/−*^ mice; (**e**) in vitro [^3^H]-cholesterol efflux assay using plasma as acceptor; (**f**) in vitro [^3^H]-cholesterol efflux assay using HDL particles as acceptor; (**g**) levels of plasma MDA as measured by assay kit; (**h**) levels of oxidized phosphocholines as measured by LC-MS/MS. CMCNa: carboxymethyl cellulose sodium. Data are expressed as the mean ± SD. & *p* < 0.05 *vs* CMCNa group; && *p* < 0.01 *vs* CMCNa group

Compared to CMCNa-treated mice, quercetin administration increased the transfer rate of [^3^H]-cholesterol from the injected macrophages to the plasma, and this was significant at the 6, 12 and 24 h time points (Fig. 1a). More importantly, compared to the CMCNa-treated mice, [^3^H]-cholesterol transported to the liver and bile for excretion was remarkably increased in the quercetin-treated mice, with 30.3% and 50.1% increases, respectively (Fig. 1b and c). As a consequence, [^3^H]-cholesterol excreted in the faeces was significantly elevated at 0–24 (35.7% increase) and 24–48 h (44.4% increase) in the quercetin-treated mice compared to the controls (Fig. 1d). Plasma and HDL from the mice were used for further investigation in vitro. The results showed that the [^3^H]-cholesterol efflux from macrophages to plasma (31.8% increase) and HDL (22.1% increase) of the quercetin-treated mice was dramatically increased compared to that of controls (Fig. 1e and f). MDA, a lipid peroxidation product, was significantly reduced in the plasma of the quercetin-treated mice compared to the CMCNa-treated mice (Fig. 1g); lipid oxidized phosphocholines, including PGPC, POVPC, (ALDO)PC, PAZPC and (COOH)PC, were significantly decreased in HDL particles from quercetin-treated mice compared with those of the controls (Fig. 1h).

### Quercetin improves cholesterol efflux and ABCA1 and ABCG1 expression in vitro

**Fig. 2:**
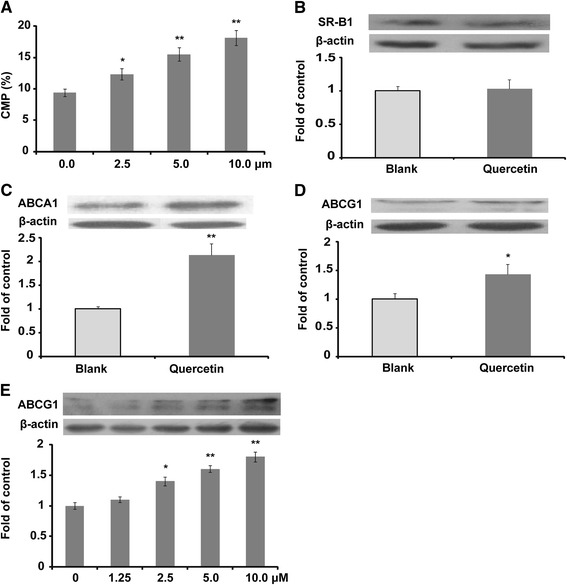
Quercetin improves cholesterol efflux and the protein expression of ABCA1 and ABCG1 in Raw264.7 macrophages (*n* = 3). **a** Quercetin improves [^3^H]-cholesterol efflux in a concentration-dependent manner; (**b**) protein expression of SR-B1 and densitometric quantification; (**c**) protein expression of ABCA1 and densitometric quantification; (**d**) protein expression of ABCG1 and densitometric quantification; (**e**) quercetin improves the protein expression of ABCG1 in a concentration-dependent manner. Data are expressed as the mean ± SD. & *p* < 0.05 *vs* blank group; && *p* < 0.01 *vs* blank group

Quercetin improved [^3^H]-cholesterol efflux in a concentration-dependent manner within a concentration range of 0 to 10 μM (Fig. 2a). Several major proteins related to cholesterol efflux were measured by western blotting, as shown in Fig. 2b-d. Quercetin significantly improved the protein expression of ABCA1 and ABCG1 at a concentration of 2.5 μM. In addition, the protein expression of ABCG1 was regulated in a concentration-dependent manner (Fig. 2e).

## Discussion

RCT is a protective mechanism involved in the development of atherosclerosis. This study reveals that quercetin significantly improves RCT in vivo for the first time using [^3^H]-cholesterol, with the underlying mechanisms possibly attributed to the increased cholesterol accepting capacity of HDL, the elevated protein expression levels of ABCA1 and ABCG1, and the reduction of oxidation. These results are helpful in clarifying the lipid-lowering effect of quercetin as previously reported [3–6].

High-fat diet induced hyperlipidaemia promotes the entry and retention of abnormal LDL within the arterial wall. The ingestion of the accumulated abnormal LDL by macrophages in the sub-endothelial space of vessels results in foam cell formation, a pathological step of early atherosclerosis. RCT could transport excess cholesterol from peripheral cells, especially macrophages, to the liver and small intestine for excretion.

In a previous study, we demonstrated that quercetin reduces hyperlipidaemia and lipid accumulation in the liver and aorta in the *apoE* ^*−/−*^ mice fed a high-fat diet [3]. It is interesting to investigate whether the lipid-lowering effect of quercetin is related to RCT. The results of this study showed that quercetin significantly improved RCT in vivo for the first time (Fig. 1). It is acknowledged that apolipoprotein A1 (apoA1) and HDL are major acceptors of peripheral cholesterol. Previously, quercetin was indicated to have no effect on the content of apoA1 [3], which is beneficial to RCT. However, the results of this study showed that quercetin treatment significantly improved the [^3^H]-cholesterol accepting capacity of the plasma from *apoE* ^*−/−*^ mice (Fig. 1e), and the increased ability may be partially attributed to the increased cholesterol accepting ability of HDL (Fig. 1f). In addition, the oxidation of HDL and apoA1 were found to reduce their cholesterol accepting ability [15]. The results of this study showed that plasma MDA and oxidized phosphocholines in HDL (Fig. 1g and h) were significantly decreased in quercetin-treated mice, and our previous data indicated that quercetin could improve the antioxidant ability of *apoE* ^*−/−*^ mice fed a high-fat diet [3]. Therefore, the antioxidant activity of quercetin may also contribute to the improved cholesterol accepting ability of HDL and apoA1.

It is well known that ABCA1 and ABCG1 mediate cholesterol efflux to apoA1 and HDL, respectively. The present study indicated that quercetin increased [^3^H]-cholesterol efflux from macrophages to media in a concentration-dependent manner in vitro. Therefore, the elevated [^3^H]-cholesterol transfer rate from [^3^H]-cholesterol-loaded macrophage to the plasma in quercetin-treated *apoE* ^*−/−*^ mice may be due to the increased protein expression of ABCA1 and ABCG1, as shown in Fig. 2, and these results are consistent with a previous in vivo study [3]. These results showing that quercetin can significantly improve ABCA1 expression are consistent with previous publications [8, 9]. It has been demonstrated that quercetin may activate ABCA1 by up-regulating PPAR-γ and LXR-α [8] or through stimulating the phosphorylation of p38 via an activation of the transforming growth factor β-activated kinase 1 and mitogen-activated kinase 3/6 [9]. In the present study, we report for the first time that quercetin could improve the protein expression of ABCG1 in a concentration-dependent manner in vitro, which is consistent with an in vivo report [3]. SR-B1 plays a significant role in the transfer of cholesterol to mature HDL. However, there were no significant changes in SR-B1 in quercetin–treated mice. A previous study from our group also showed that the ethanolic extract of propolis (quercetin is one the major effective components of propolis) has no significant influence on the protein expression of SR-B1 both in vitro and in vivo [11]. However, the effect of flavonoids on SR-B1 protein expression remains unresolved.

In mice, cholesterol can be delivered to liver directly by SR-B1 and indirectly by the LDL receptor after free cholesterol has been transformed to cholesterol esters by the action of lecithin cholesterol acyltransferase. In other words, cholesterol esters of very low density lipoprotein and LDL could be removed by the liver through the LDL receptor, which is beneficial to RCT. In a previous study, quercetin was demonstrated to increase the protein expression of the LDL receptor in vivo [3], which could partially explain the elevated liver [^3^H]-cholesterol in *apoE* ^*−/−*^ mice in the present study. Therefore, quercetin may elevate the liver uptake of [^3^H]-cholesterol mainly by up-regulating the LDL receptor pathway in *apoE* ^*−/−*^ mice. Furthermore, ABCG5 is an important mediator involved in the secretion of cholesterol from the liver and small intestines. Quercetin treatment up-regulates the expression of ABCG5 in liver and small intestine [3], which may contribute to the increase of [^3^H]-cholesterol in the bile and subsequently in the faeces of *apoE* ^*−/−*^ mice. As one of the widely-distributed flavonoids in plants, quercetin is also one of the major components of natural nutraceuticals. Therefore, quercetin may partially contribute to the lipid-lowering effect of nutraceuticals, as has been well documented by Scicchitano et al. [16].

Plasma cholesteryl ester transfer protein (CETP) facilitates the transfer of cholesteryl esters from HDL to LDL and VLDL and is a key protein in RCT. However, mice naturally lack CETP. Furthermore, mouse plasma is characterized as HDL-C dominant, while human plasma is non-HDL-C dominant. Therefore, the major limitation of the present study is that the results may not reflect what is occurring in humans. Thus, our next plan is to verify the effect of quercetin in CETP transgenic mice, whose lipid profile is closer to humans than *apoE* ^*−/−*^ mice. Another limitation of this study is that the pathways are not further investigated with the help of interfering means such as siRNA.

## Conclusions

Quercetin improves RCT by up-regulating related protein expression levels, such as ABCA1 and ABCG1, and also by elevating the cholesterol accepting ability of HDL and apoA1 via reducing oxidation.
